# Loneliness and preferences for objects in light versus dark background lighting

**DOI:** 10.3389/fpsyg.2026.1700055

**Published:** 2026-02-12

**Authors:** Fuschia M. Sirois, Yanan Wang

**Affiliations:** 1 Department of Psychology, Durham University, Durham, United Kingdom; 2 Williams School of Business, Bishop’s University, Sherbrooke, QC, Canada

**Keywords:** background lighting, conceptual metaphor, decision-making, embodied cognition, loneliness, self-schemas

## Abstract

**Introduction:**

When presented with temperature related options that metaphorically reflect loneliness or non-loneliness, lonely people choose the non-lonely option. Less is known about the reasons for this preference, or whether it also extends to the visual domain. Across six experiments (Total *N* = 1,725) we investigate whether background lighting that evokes a conceptual metaphor of loneliness activates undesired self-schemas and motivates preferences for objects presented with bright versus dark background lighting.

**Methods and results:**

Studies 1-2 found that chronic and state loneliness were associated with preferences for objects in brightness rather than darkness. Study 3 replicated results from Study 2 and provided evidence for the idea that lonely people do not prefer objects in darkness because they evoke a negative self-congruity with the object. Study 4 demonstrated that engaging in conscious information processing of dark background lighting eliminated the effects of loneliness on object preference and the associated negative emotions. Studies 5 and 6 provided evidence that the effects of loneliness were only for objects that had self-referent salience, supporting an ideal self-object congruity hypothesis.

**Conclusion:**

This research reveals a novel link between loneliness and lighting preferences, and as such advances understanding of the metaphorical mapping of loneliness, and the implications of individual differences in loneliness for decision-making and consumer behavior.

## Introduction

Loneliness has been defined as the subjective feeling of social isolation that involves feelings of social pain from not having social needs met (Hawkley and Cacioppo, 2010; Cacioppo et al., 2006). As such, loneliness implies a failure to successfully connect with others on a social level, which is reflected in the ways that lonely people are perceived. The social stigma associated with loneliness (e.g., Barreto et al., 2022) means that lonely people are viewed as having a number of undesirable characteristics, such as being less sociable (Rotenberg et al., 2002), and less warm and understanding, less confident, and less trusting of others (Tsai and Reis, 2009). When people feel lonely they not only view themselves more negatively (Jones et al., 1981), but they also tend to view the world differently. For example, lonely people pay more attention to negative social stimuli (Cacioppo et al., 2015), and are more sensitive to negative social cues in their environment (Cacioppo et al., 2014).

Theory and evidence suggest that the preferences of people who are lonely are influenced by a sensitivity to negative and self-relevant aspects of the environment. Consistent with an embodied view of cognition (Barsalou, 2008), when presented with options that metaphorically reflect loneliness (cold food) or non-loneliness (warm food), people who are lonely show a preference toward the non-lonely option (Zhong and Leonardelli, 2008). With loneliness increasing to alarmingly high rates in recent years in the United Kingdom (Office of National Statistics, 2025), North America (Demarinis, 2020), and globally (Barreto et al., 2021), and after the pandemic (Palgi et al., 2020), understanding how and why loneliness might influence people’s judgments, decisions, and behavior is becoming increasingly important.

In the current paper we extend our understanding of how lonely people perceive and interact with their environment by investigating the effects of loneliness on preferences and decision-making as a function of one particular sensory experience of the environment, namely visual darkness levels. We propose that the metaphorical associations of loneliness with object temperature found in previous research (Zhong and Leonardelli, 2008) extends to visual perceptions of environmental lighting, and that darkness, a metaphor often linked to loneliness, might also influence lonely people’s preferences for objects presented with lighting reflecting loneliness (darkness) versus non-loneliness (brightness). In addition, we investigate whether lonely people’s preferences for non-lonely lighting options are motivated by a desire to avoid being personally associated with the lonely option because this can heighten feelings of loneliness and thus threaten self-esteem.

### Individual differences in loneliness

Loneliness can be conceived of as a stable individual difference variable or as transient feelings of social isolation. From an evolutionary perspective, loneliness can be functional in that the unpleasant feelings from being lonely promote survival by prompting seeking out meaningful social connections, which can in turn reduce feelings of loneliness (Cacioppo et al., 2014). However, activation of this “affiliation motive” (Qualter et al., 2015) is not always successful, especially for individuals with negative cognitive biases. Those who are hypervigilant to social cues and/or prone to interpreting such cues as being threatening, may instead elicit behaviors from others that confirm their feelings of isolation, leading to a self-reinforcing loop of loneliness that can become chronic over time (Cacioppo and Hawkley, 2009).

The nomological network of loneliness confirms this account of how loneliness can become a stable trait-like quality as well as its linkages to negative cognitive biases. Behavioral genetics studies have found that loneliness has the largest phenotypic, genetic and environmental correlations with Neuroticism, even after accounting for the contributions of the remaining Big five personality factors (Schermer and Martin, 2019). Loneliness is also associated with lower Conscientiousness, Extraversion, and Agreeableness (Flett et al., 2016; Schermer and Martin, 2019; Cacioppo et al., 2006). With respect to other traits and states, higher loneliness is associated with higher negative mood, anxiety, fear of negative evaluation, and avoidant thinking, and lower levels of mattering (Flett et al., 2016), optimism, social skills, positive mood, and self-esteem (Cacioppo et al., 2006). Importantly, experimental research manipulating loneliness found corresponding changes in these states and traits (Cacioppo et al., 2006), supporting the proposition that loneliness is more than feeling unhappy, and involves heightened sensitivity to attacks and threats (Cacioppo et al., 2014).

### Embodied cognition and loneliness

Embodied cognition (Barsalou, 2008), is one theoretical framework that can provide insights into why loneliness might be associated with visual darkness. Embodied views of cognition are based on the idea that cognitive processes are grounded in the body’s interaction with the world and that these links are bidirectional. Accordingly, individuals come to understand the world around them from an integrated body–mind perspective, with sensory experiences informing mental states, and vice versa (Barsalou, 2008). Research has demonstrated embodiment effects across all five senses. For example, people who ate sweets behaved in a more pro-social or “sweet” manner (Meier et al., 2012), those exposed to fishy smells reported being more suspicious (Lee et al., 2015), others are perceived as being socially warmer after holding a warm rather than a cold cup of coffee, heavier books are evaluated as being more important than lighter ones (Chandler et al., 2012), and tourists rated their experiences as being psychologically darker for images and settings that had greater visual darkness versus brightness (Lv et al., 2022).

It is worth noting that embodied cognition research has attracted both theoretical (Goldinger et al., 2016), and methodological criticisms (Wortman et al., 2014). For example, Wortman et al. (2014) did not find the increases in loneliness after holding a cold versus warm pack that were reported by Bargh and Shalev (2012), effectively failing to replicate the connection between physical and interpersonal coldness. Yet despite these criticisms, there is a substantial and growing body of research that supports embodied effects and their application across a number of different life domains (Farina, 2020).

With respect to loneliness, research into its embodied effects is limited. Across two experiments, individuals who were induced to feel lonely estimated lower room temperatures and showed a greater preference for warm food and drink because they felt colder (Zhong and Leonardelli, 2008), supporting the idea that loneliness is embodied as the psychological experience of coldness. Similarly, higher scores on a measure of chronic loneliness were associated with a greater tendency to take warm baths and showers, and manipulating physical coldness resulted in greater feelings of loneliness (Bargh and Shalev, 2012). There is also some evidence that the embodied coldness associated with loneliness extends to identification with protagonists in the media. Viewers of a lonely protagonist in a film expressed greater identification with this character when they were in a cold versus a warm room (Tal-Or and Razpurker-Apfeld, 2021b). Similarly, media consumers who identified with media characters who were lonely or physically cold experienced the corresponding embodied states of physical coldness or psychological loneliness (Tal-Or and Razpurker-Apfeld, 2021a).

Although there has been little embodied cognition research on loneliness outside of the temperature domain, it is possible that these embodied effects of loneliness also extend to the visual domain. Environmental darkness, which can be characterized as the absence of light and therefore warmth, might similarly embody loneliness. Evidence from the embodied effects of a related concept, social exclusion, supports this proposition. Across four studies, participants who were socially excluded perceived objects and their environments to be visually darker than they were (Pfundmair et al., 2019). From a theoretical perspective, cognitive linguistic models of embodied cognition suggest that people draw upon their knowledge of their physical and bodily experiences to metaphorically ground abstract concepts (Barsalou, 2008). Thus, people may associate loneliness with darkness based on its various metaphorical connotations.

### Darkness as a conceptual metaphor for loneliness

A number of everyday metaphors and literary references suggest an association between loneliness and the concept of darkness. Phrases such as “left in the dark” and “in the shadows” are often used to communicate social exclusion and isolation, states which are conceptually linked to loneliness. In Robert Frost’s poem “Acquainted with the Night,” the night (a time of darkness) is used as a metaphor for loneliness and depression. In H. G. Well’s “The Time Machine,” the main character’s fear of the darkness of the well is amplified by knowing that he is all alone: “But I was so horribly alone, and even to clamber down into the darkness of the well appalled me.” Taking a more concrete perspective, being in darkness literally means that people cannot see their surroundings nor if anyone else is nearby, which can be analogous to feeling alone and isolated.

Conceptual metaphor theory (Lakoff and Johnson, 1980) provides one explanation as to how darkness and feelings of loneliness may become metaphorically linked. This theory suggests that our understanding of higher level, abstract concepts is informed by our lower level more concrete experiences in the world, such that the easily grasped lower concepts (e.g., darkness) are used to gain a better understanding of higher-level concepts (e.g., loneliness) for which we may have relatively less experience and knowledge. Indeed, a number of conceptual metaphors are grounded in physical embodied experiences (Barsalou, 2008). Although to date there is no research that we are aware of that directly examines the visual embodiment effects of loneliness, evidence on related constructs supports the proposed metaphorical links between loneliness and darkness. In addition to the research on social exclusion and judgments of darkness described previously (Pfundmair et al., 2019). Dong et al. (2015) found that induced hopelessness, an emotional state that is strongly associated with loneliness (Chang et al., 2010; Joiner and Rudd, 1996; Ruchkin et al., 1999), resulted in perceiving a room to be darker than it was, which in turn increased desire for a brighter room. There is also experimental evidence demonstrating that dark ambient lighting makes people feel socially disconnected from others (Huang et al., 2018), and can enhance feelings of anonymity (Zhong et al., 2010). Considering this theory and research, a metaphorical connection between loneliness and darkness is plausible.

### Self-schema activation

The above research has demonstrated the literal translation of the metaphors associated with the negative states of loneliness and hopelessness into metaphorically-linked sensory and perceptual changes. This research together with theory on embodied cognition imply that feelings of loneliness automatically evoke conceptual metaphors that may affect people’s preferences in different ways. For example, it is possible that loneliness may influence people’s preferences for objects that are presented in ways that metaphorically reflect loneliness. Following this logic, objects presented against a backdrop of dark lighting may be viewed by lonely people as being consistent with their own emotional state, which could have the effect of heightening awareness of feeling lonely, a state which is unpleasant to most people (Hawkley and Cacioppo, 2010). This proposition is consistent with self-product congruency theory (Sirgy, 1982, 1985), which proposes that products (a specific class of objects) can serve as cues that activate self-schemas about actual self-image (who a person sees themselves as now), and ideal self-image (who a person would like to be). From this perspective, objects that are congruent with actual self-image reflect self-congruity, whereas objects that are congruent with ideal self-image reflect ideal-congruity.

Research has demonstrated that self-congruity, whether with the actual or ideal self, can impact consumer behavior. For example, self-congruity with a positive self-image, such as being someone who engages in ecotourism, increases willingness to pay more for ecotourism (Moons et al., 2020). Self-congruity can also affect not only purchasing behavior (Ericksen and Sirgy, 1992), but also brand loyalty (Kressmann et al., 2006). Although either form of self-product congruity can motivate preferences for a product, the nature of the preference depends also on how both the self and the product are evaluated. Sirgy (1985) proposes that the relative balance of the self-esteem motive (a need to act in ways that enhance self-esteem) and the self-consistency motive (a need to act in ways consistent with self-image) are key for understanding how self-product congruity influences product preferences when self-image is negative or positive. For someone who is lonely and has a self-image that is negative, a product in dark lighting that evokes the conceptual metaphor of loneliness, would also be viewed as negative. This negative self-congruity would result in a product preference conflict because motivation to enhance self-esteem would promote product avoidance, whereas motivation to maintain self-consistency would promote product approach or preference. However, we argue that because loneliness has consistently been found to be associated with low self-esteem (McWhirter, 1997; Olmstead et al., 1991; Vaux, 1988; Cacioppo et al., 2000), the motivation to self-enhance may outweigh the motivation for self-consistency. Accordingly, we propose that lonely people will avoid choosing an object presented in darkness because it evokes a negative self-schema and associated negative feelings.

Sirgy (1985) has also argued and demonstrated that people who are low in self-esteem tend to be more influenced by ideal-congruity in their product preferences, as products that activate ideal self-schemas offer the promise of seeing oneself in a positive light and are therefore self-enhancing. If we consider that brightness is the absence of darkness, then objects presented with bright background light may be similarly viewed as metaphorically reflecting the absence of loneliness (see Dong et al., 2015 for an analogous argument regarding hope and hopelessness), and therefore be viewed as desirable by people when they feel lonely.

### The present research

Taken together, this theory and research provides support for the proposition that when people who feel lonely are presented with the choice of an object with dark versus bright background lighting, they will show a preference for the object with bright lighting. This proposition is consistent with both self-product congruency theory (Sirgy, 1982, 1985), and evolutionary perspectives on loneliness which posit that lonely people have negative cognitive biases that make them sensitive to threatening cues in the environment (Cacioppo et al., 2014; Cacioppo and Hawkley, 2009; Qualter et al., 2015). The preference for objects in light versus dark background lighting could reflect either approach or avoidance motivations. The latter would be indicated by a congruity between the lonely person’s ideal self-image and the object, such that the presentation of the object in brightness reflects a desired self that enhances self-esteem, or by wanting to avoid the congruity between actual (lonely) self and the object presented in darkness, because this is threatening to self-esteem (Sirgy, 1985). We propose that because loneliness is associated with low approach and high avoidance motivations (Mehrabian, 1994), and avoidant thinking (Cacioppo et al., 2006), the latter may more accurately reflect the motivations that underlie the proposed preference for objects presented in bright versus dark ambient lighting when people feel lonely. Lastly, because objects in dark background lighting imply being surrounded by darkness (and by extension, feelings of loneliness), we argue that it is the darkness of the background rather than the darkness of the object that may evoke feelings of loneliness.

Previous research has found that simulating light and dark ambient lightning via differences in background lighting can evoke the embodied effects of vision. In a study on dark tourism, Lv et al. (2022) found that altering darkness on a two-dimensional plane (i.e., within a photo) successfully simulated differences in dark and light ambient lighting and influenced psychological states in a manner similar to that when actual visual levels of three dimensional lightness/darkness were changed. Accordingly, we used changes in object background lighting as a proxy for changes in three-dimensional visual lightness versus darkness.

We tested these hypotheses across six experiments, employing different types of common consumer objects (furniture, T-shirts, water bottles, coffee mug, toothbrush, and eyeglasses) and both dark and light-colored objects, with measures of both trait and state loneliness, positive and negative feelings toward the objects presented in dark versus bright background lighting, and ruling out confounding variables and competing hypotheses. We chose everyday products that people might consider buying as the target objects to assess object preferences. An overview of the aims and designs of the six studies is presented in Table 1.

**Table 1:** Overview of the aims and designs of the six studies.

Study	Study aims	Study design	Type of loneliness	Type of objects
1	Test the effects of loneliness on preferences for objects presented with either dark or bright background lighting, controlling for depression, and object color.	Within subjects	Chronic loneliness	Desk, shelving unit
2	Replicate effects of Study 1 with loneliness manipulation.	Between subjects	State loneliness (induced)	Desk, shelving unit
3	Replicate effects of Study 2 and test the role of negative emotions for product preferences.	Between subjects	State loneliness (induced)	Desk
4	Directly test the effects of dark background lighting of products on emotional experience of loneliness, by making them more salient to eliminate the effects from Study 3.	Between subjects	State loneliness (induced)	Desk
5	Test the role of self-schema in object preferences by comparing preferences for objects for self- versus others.	Mixed between-within subjects	Chronic loneliness	Gender-neutral T-shirt, coffee mug, electric toothbrush
6	Replicate Study 5 to test the role of object functionality by comparing object wearability.	Between subjects	Chronic loneliness	Water bottle, pair of sunglasses

## Study 1

Our first study tested the effects of chronic loneliness on preferences for objects presented with either dark or bright background lighting. To rule out the possibility that the effects were due to the darkness or lightness of the object itself rather than the background lighting, we also tested the effect of loneliness on preferences for objects with dark and light colors. Because loneliness is associated with, but distinct from, depression (Marroquín et al., 2016; Hawkley and Cacioppo, 2010; Cacioppo et al., 2006), and depression might influence sensitivity to darkness (Cawley et al., 2013), we included a measure of depression in the questionnaire to rule out depression as an alternative explanation for our findings. Depression was analyzed first as an independent variable predicting product preferences, and then as a covariate to control for its potential effects on product background lighting preferences.

### Method

Approval from the Bishop’s University Research Ethics Committee (Canada) was received prior to data collection. Participants’ consent was obtained online via indicating agreement to an online consent form administered prior to data collection. No power analysis was conducted *a priori*. The aim was to recruit as many participants as possible with the budget limits. Data was collected in May 2012.

#### Participants

A sample of 228 undergraduates (52.2% female; Mean age = 19.5, *SD* = 2.82) at a Canadian university voluntarily participated in the study in exchange for three dollars.

##### Procedure and materials

**Figure 1:**
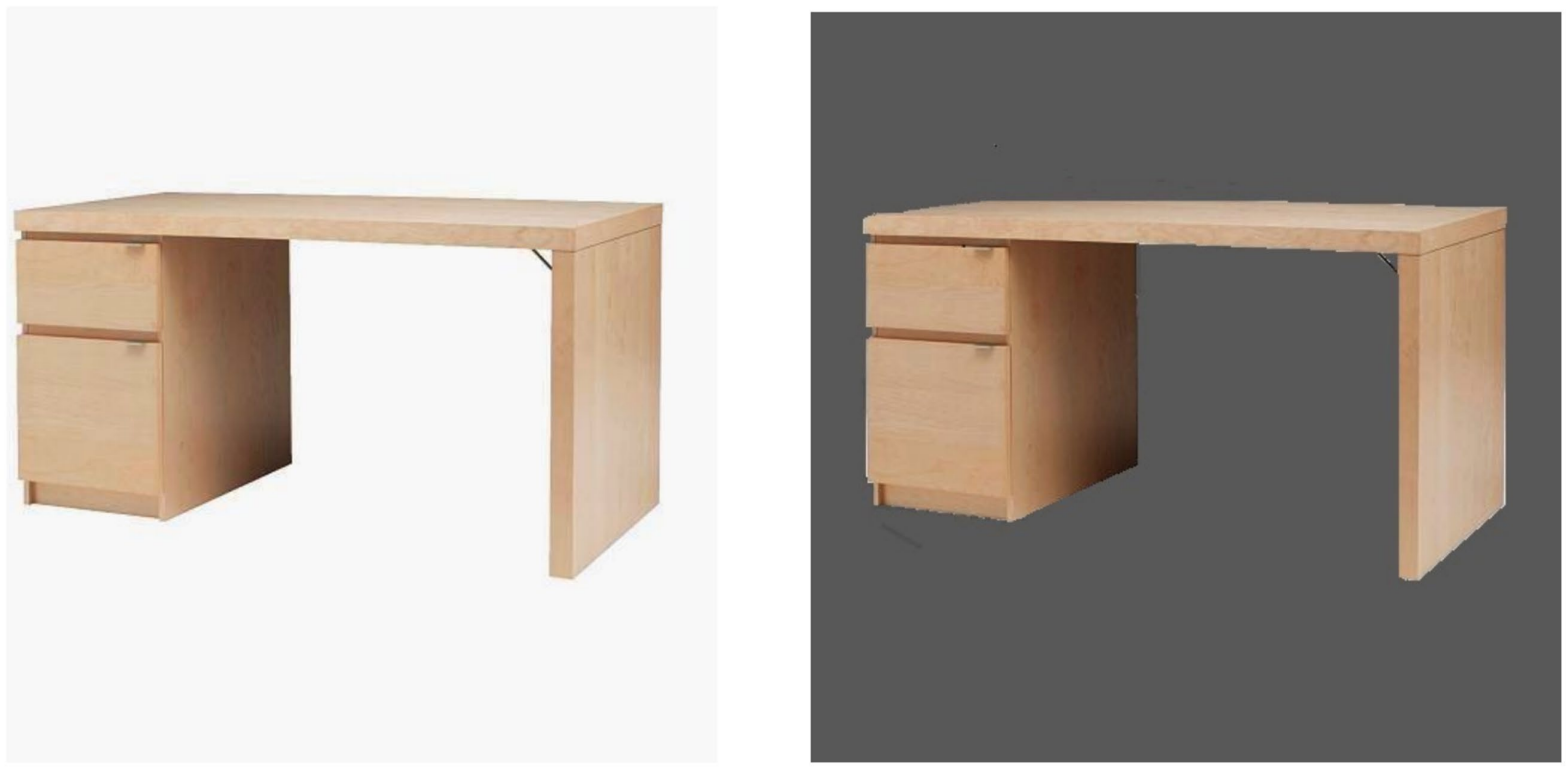
Desk presented with bright and dark background lighting.

**Figure 2:**
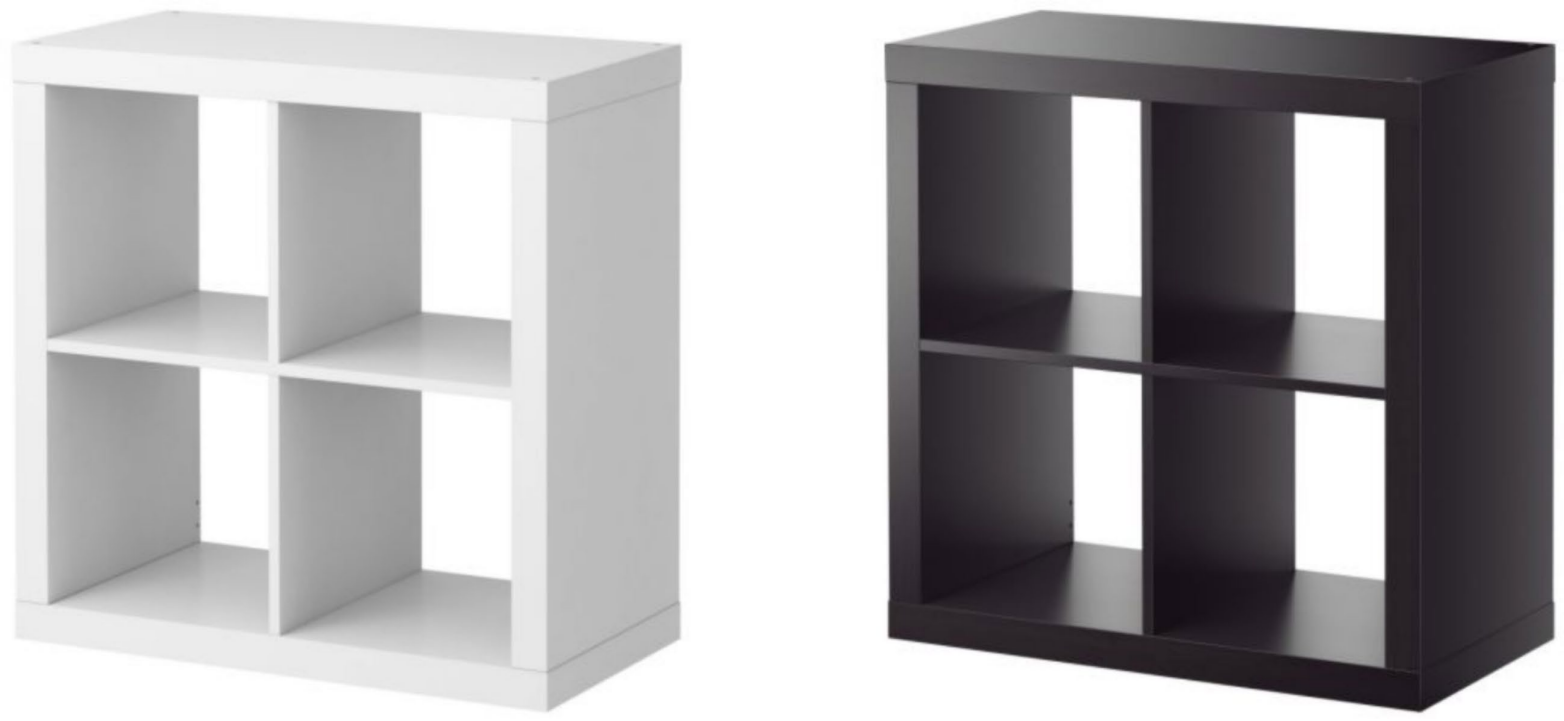
Light and dark shelving units.

Participants were first asked to answer filler questions from the Big Five Personality test (John et al., 1991). They were then presented two different IKEA product pairs. The first image featured a beige computer desk presented with the background as either in bright or dark lighting (Figure 1), to test the main hypothesis. The second image presented a shelving unit with the product color as either black or white (Figure 2), to rule out the confounding effect of product color. After viewing the product images, participants indicated their product preference across three items: (1) How do you like the product? (2) How interested are you in the product? (3) How likely are you to get this product in the future? Items were rated on a 7-point scale (1 = *not at all*; 7 = *very much*), for both the desk (*α* = *0*.90) and the shelving unit (*α* = 0.89). Participants completed the 20-item revised UCLA loneliness scale (Russell, 1996), and the Beck Depression Inventory (BDI; Beck et al., 1996). Items on the loneliness scale are rated from 1 (*never*) to 4 (*often*), and summed after reverse coding appropriate items, with higher scores reflecting greater feelings of loneliness (*α* = 0.92). The BDI includes 20 items rated on a 0–3 point scale, which are summed to obtain a total depression score (*α* = 0.84).

We categorized participants as *lonely* versus *non-lonely* based on the established cutoff of the original UCLA loneliness scale. The scale consists of 20 items rated 1–4, yielding a total possible score from 20 to 80. Following the standard convention for this measure, a total score of 40 or below indicates *non-loneliness*, whereas a total score of 41 or above indicates *loneliness*. Using this cutoff, 115 participants fell into the non-lonely group, and 113 participants fell into the lonely group. For our data analyses, we used mean-centered loneliness scores. When expressed in mean-centered units, the cutoff corresponded to a score of −1.23 or below for the non-lonely group and −0.23 or above for the lonely group. These values are the centered equivalents of the original cutoff of 40/41.

### Results and discussion

**Figure 3:**
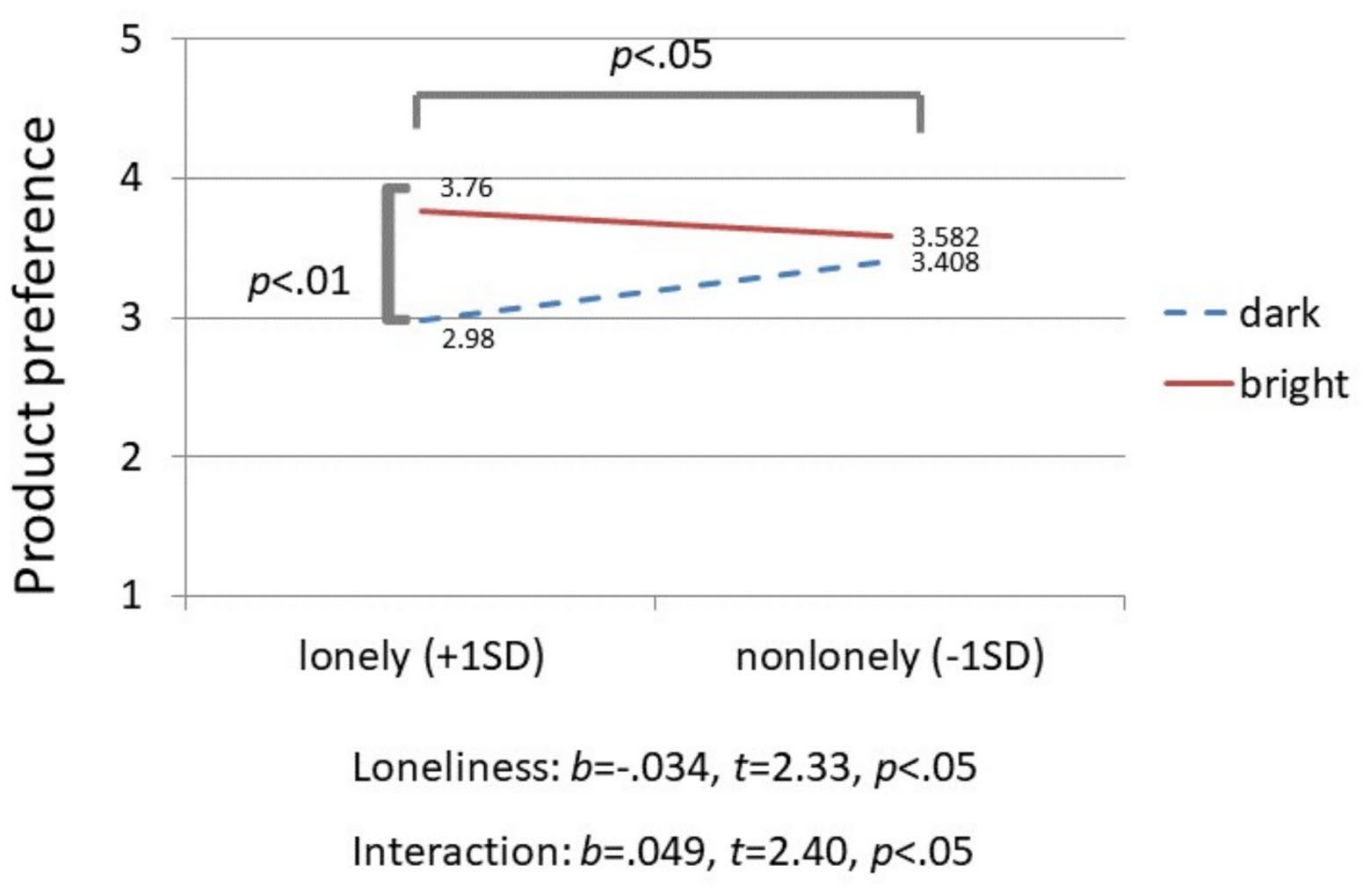
Spotlight analyses at 1 standard deviation above and below the mean of loneliness scores testing the interaction between loneliness and background lighting on product preferences in Study 1.

To test our hypotheses, we conducted a regression analysis with product preference toward the computer desk as the dependent variable and loneliness (mean-centered), background lighting (bright versus dark), and their interaction, as predictors. Results showed a significant main effect of loneliness (*β* = −0.034, *t* = 2.33, *p* < 0.05) and a significant interaction effect (*β* = 0.049*, t* = 2.40, *p* < 0.05). We then conducted simple slope analyses of the effects of loneliness on product preference as a function of background lighting. The results revealed that lonely (vs. non-lonely) participants reported significantly lower product preference when the background lighting was dark (*β* = −0.034*, t* = −2.38, *p* < 0.05*, ηp^2^* = 0.05) than when it was bright (*β* = 0.014, *t* = 0.926, *p* = 0.35, *ηp*^2^ = 0.007). We then conducted spotlight analyses at 1 standard deviation above and below the mean of loneliness scores to examine the interaction between loneliness and background lighting from a different perspective (see Figure 3). A total of 145 participants (63.5% of the sample) fell within ±1 SD range (SD = 9.79). The results were consistent with the ideal-self congruency hypothesis in that lonely participants reported higher preference when the desk’s background was bright than when it was dark (*β* = 0.488, *t =* 2.65, *p* < 0.01), whereas non-lonely participants’ preference toward the desk did not vary as a function of its background lighting (*β* = −0.118, *t* = 0.64, *p* = 0.52).

To rule out the confounding effect of product color, we also conducted a regression analysis with product preference toward the shelving unit as the dependent variable and loneliness (mean-centered), product color (0 = *black*; 1 = *white*), and their interaction as predictors. There was no significant main effect of loneliness and product color (*p’s* > 0.20), or their interaction effect (*p’s* > 0.90), supporting our assertion that it was product background lighting, and not product color, that impacted participants’ preferences.

To rule out depression as an alternative explanation for our findings, we first ran a regression analysis with depression, background lighting, and their interaction as predictors of product preference. None of the variables were significant predictors (*p’s* > 0.10). We then ran a regression with loneliness, depression, background lighting, and all the interactions as independent variables. Results showed that neither the main effect of depression nor its interactions with any other variables were significant (*p’s* > 0.20). We next analyzed our data by controlling for depression and found that all previously found effects of loneliness (*β* = −0.025, *t* = 2.52, *p* < 0.05) and its interaction with background lighting (*β* = 0.038, *t* = 2.80, *p* < 0.05) remained essentially unchanged, and the effect of depression on product preference was not significant (*p* = 0.82). Consistent with previous research indicating that loneliness and depression are related but distinct (Cacioppo et al., 2006), these findings suggest that loneliness affected participants’ preference for the background lighting of the desk independent of depression.

## Study 2

Our second Study was designed to expand the findings from Study 1 in two key ways. Experiment 1 examined the effects of dispositional loneliness on product preferences in a student sample. In Experiment 2 we aimed to replicate the findings from Study 1 with a general, non-student population. Previous experimental research has found that loneliness can be manipulated and that those who are induced to feel lonely momentarily display many of the attributes and characteristics of those who are chronically lonely (Cacioppo et al., 2006). Accordingly, we also tested whether the effect of state loneliness on product preferences would be the same as those found for dispositional loneliness using a between-subjects design. To test this, participants were randomly assigned to either a lonely or a non-lonely essay writing task designed to manipulate state loneliness, before asking them to rate their product background lighting preferences as per Study 1.

### Method

Approval from the Bishop’s University Research Ethics Committee (Canada) was received prior to data collection. Participants’ consent was obtained online via indicating agreement to an online consent form administered prior to data collection. No power analysis was conducted *a priori*. The aim was to recruit as many participants as possible within the budget limits. Data was collected in November 2014.

#### Participants

A total of 399 adults (48.6% female, 67.8% aged between 25 and 44 years old) recruited from Mechanical Turk participated in an online experiment in exchange for 75 cents.

#### Procedure and materials

Participants completed the same personality filler questions as in Experiment 1. They were then asked to provide answers to an open-ended question in a recall task. Participants in the state loneliness condition wrote an essay in response to instructions based on previous research that induced loneliness (Cacioppo et al., 2006): “*Please write an essay about a time in which you felt isolated; you felt lonely; perhaps you felt like you just did not belong—that you had no friends*.” Participants in the non-lonely condition wrote an essay in response to: “*Please write an essay about a time in which you felt a sense of belonging; perhaps you were a member of a group; perhaps you had a best friend with whom you felt you could share anything.”* Participants then answered a self-perception scale, which included two items that served as a manipulation check for state loneliness: “I am lonely” and “I am isolated from others” (*α* = 0.85), rated on a 1–7 Likert scale (1 = *not at all*; 7 = *very much*). They were then presented with two images in a counterbalanced order featuring the IKEA desk and shelving unit as in Experiment 1, with the product background in a bright or dark ambient lighting (Figures 1, 2).

### Results and discussion

The loneliness manipulation was successful, with participants in the loneliness condition reporting significantly higher levels of loneliness (*M* = 3.48, *SD* = 1.74) than those in the control condition (*M* = 2.80, *SD* = 1.71; *F* (1, 397) = 14.60, *p* < 0.001). The between-subjects ANOVA with preference toward the computer desk as the dependent variable and loneliness (non-lonely versus lonely condition), background lighting (bright versus dark), and their interaction as predictors, revealed a significant interaction effect between loneliness and background lighting (*F* (1,397) = 12.15, *p* < 0.001, *ηp*^2^ = 0.03). However, the main effects of state loneliness (*F* (1, 297) = 1.615, *p* = 0.20), and background lighting (*F* (1, 297) = 0.006, *p* = 0.90) were not significant. Pairwise comparisons using the overall error term showed that participants in the lonely condition reported significantly higher preference toward the computer desk when its background lighting was bright (*M* = 3.29, *SD* = 1.92) than when it was dark (*M* = 2.63, *SD* = 1.57, *F* (1, 196) = 6.70, *p* < 0.01, *ηp*^2^ = 0.033). In contrast, participants in the non-lonely condition reported a preference toward the computer desk when its background lighting was dark (*M* = 3.34, *SD* = 1.96) versus when it was bright (*M* = 2.86, *SD* = 1.81, *F* (1, 199) = 3.18, *p* > 0.05, *ηp*^2^ = 0.016) that did not differ significantly. Overall, the findings were consistent with those in Experiment 1, and the ideal-self congruity hypothesis in that participants made to feel lonely preferred an object with bright background lighting.

As in Experiment 1, we again tested the potential confounding effect of product color. The ANOVA revealed no significant main effect for loneliness (*F* (1, 393) = 0.23, *p* = 0.63), product color (*F* (1, 393) = 0.03, *p* = 0.86), or the interaction effect (*F* (1, 393) = 0.13, *p* = 0.72). This supported the notion that it was product background lighting, and not product color, which impacted the product preference of those in the lonely condition.

## Study 3

The purpose of Study 3 was to replicate the results from Study 2, and to further test the role of negative emotions in explaining lonely participants’ non-preference for objects in dark background lighting. Consistent with conceptual metaphor theory (Lakoff and Johnson, 1980), and self-product congruency theory (Sirgy, 1982), we posited that products that have a dark background lighting that simulated environmental darkness may evoke the conceptual metaphor of loneliness and be viewed by lonely people as being congruent with their own emotional state. This in turn would create a negative self-congruity with the product, activate a negative self schema, and result in product avoidance to enhance self-esteem. Accordingly, we expected that products with a dark background lighting would elicit negative feelings for lonely participants that would explain their non-preference for products in dark background lighting. To rule out the possibility that the non-preference for the product presented in dark background lighting was due to less positive feelings when viewing that product, or a positive feeling when viewing the product presented in bright background lighting, we also asked participants to rate their positive emotions toward the products. We then ran bootstrapped indirect effects analyses with 5,000 resamples for both negative and positive product emotions.

### Method

Approval from the Bishop’s University Research Ethics Committee (Canada) was received prior to data collection. Participant consent was obtained online via indicating agreement to an online consent form administered prior to data collection. Data was collected in May 2015.

#### Participants

A total of 232 participants (61.5% female; 66.1% aged from 25 to 44 years old) recruited via Mechanical Turk participated in an online experiment in exchange for $1.30. No power analysis was conducted *a priori*. The aim was to recruit as many participants as possible within the budget limits ($300).

#### Procedure and materials

Similar to Study 2 participants were randomized to either a loneliness or non-loneliness condition, and completed the personality filler questions, a writing task, and manipulation check items. They were then presented the images of the desk (Figure 1) as in Study 1 and asked to rate the emotions experienced when viewing the desk using the PANAS scale (Watson et al., 1988), a 20-item measure of current positive (10 items; *α* = 0.94) and negative (10 items; α = 0.91) affective states which are rated from 1 (*very slightly or not at all*) to 5 (*extremely*). This measure assessed the extent to which participants experienced negative feelings versus positive feelings about the desk when presented with dark background lighting may explain the association between loneliness and product preferences. Participants then rated their preferences for the product using the four-item scale (α = 0.96) from Study 1.

### Results and discussion

Participants in the loneliness condition reported significantly higher level of loneliness (*M* = 3.15, *SD* = 1.92) than those in the control group (*M* = 2.57, *SD* = 1.79; *t* (229) = 5.63, *p* < 0.05), indicating that the loneliness manipulation was again successful.

The ANOVA revealed a significant interaction effect between loneliness and background lighting (*F* (1, 227) = 6.83*, p* < 0.01, *ηp^2^ = 0*.029). As in Experiment 2, there was no significant main effect of state loneliness (*F* (1, 227) = 0.411, *p* = 0.52) or background lighting (*F* (1, 227) = 1.92, *p* = 0.17). Pairwise comparisons showed that participants in the lonely condition reported significantly higher preference toward the computer desk when its background lighting was bright (*M* = 3.88, *SD* = 1.61) than when it was dark (*M* = 2.95, *SD* = 1.66, *F* (1, 115) *=* 9.21, *p* < 0.01, *ηp*^2^ = 0.074). In contrast, participants in the non-lonely condition did not report significantly different preference toward the computer desk when its background lighting was dark (*M* = 3.70, *SD* = 1.91) or when it was bright (*M* = 3.42, *SD* = 1.80, *F* (1, 112) = 0.66, *p* = 0.42, *ηp*^2^ = 0.006). Overall, the results were generally consistent with those in Studies 1 and 2 and demonstrated that participants who felt lonelier preferred objects with bright rather than dark, background lighting.

Consistent with our negative self-congruity hypothesis, the *t*-test revealed that lonely participants reported significantly higher levels of negative emotion toward the product when it was presented with a dark (*M* = 4.44, *SD* = 1.78) compared to a bright (*M* = 3.51, *SD* = 1.80, *t* (114) = 7.78, *p* < 0.01), background lighting. However, the ratings for positive emotion toward the product presented in dark (*M* = 3.05, *SD* = 1.80) versus bright (*M =* 2.52; *SD* = 1.66, *t* (114) *=* 2.84, *p* = 0.09) background lighting were not significantly different, suggesting that the non-preference of the products in dark background lighting was driven by avoidance of the negative product-related feelings rather than preference for the positive product-related feelings associated with the products in bright background lighting.

A bootstrap analysis of the indirect effects of loneliness on product preference via negative emotion for the participants in the loneliness condition was significant (*b =* −0.75, *t* = −12.23, *p* < 0.001; 95% CI: [−1.22, −0.24]), indicating that higher levels of negative affect for the desk presented with dark background lighting accounted for the lower product preference ratings. However, the test of the indirect effects of positive emotion was not significant (*p* > 0.30), nor were any of the tests when repeated for the non-lonely participants (*p’s* > 0.20). Taken together these findings support the notion that a negative self-congruity between loneliness and products presented in dark background lighting explain participants” non-preference for these products.

## Study 4

Studies 1 to 3 demonstrated that loneliness is associated with a preference for products presented with bright rather than dark background lighting, and Study 3 provided support for the idea that the non-preference of products in dark background lighting is due to negative feelings toward these products which evokes a negative self congruity with the product. In Study 4 we sought to more directly test the proposition that products presented with dark background lighting are metaphorically linked to the emotional experience of loneliness, and that presentation of such products automatically evokes a conceptual metaphor of loneliness. This proposition is consistent with previous research on embodied cognition within the temperature domain which demonstrated that people are not explicitly aware of the association between physical and social coldness, suggesting that embodied effects reflect a self-regulatory process that is unconscious (Bargh and Shalev, 2012). Because semantic network models of emotion (Bower, 1981; Ingram, 1984) suggest that the activation and processing of information nodes related to an emotion is largely an automatic rather than a conscious process, we asked participants to consciously think and write about the product presented with dark ambient lighting to test the hypothesis that the metaphoric effects of darkness on object preference are processed through an unconscious rather than a conscious route. We expected that doing so would eliminate the indirect effects of loneliness on product preference through negative emotions that were tested and supported in Experiment 3.

### Method

Approval from the Bishop’s University Research Ethics Committee (Canada) was received prior to data collection for this between subjects study. Participants’ consent was obtained online via indicating agreement to an online consent form administered prior to data collection. No power analysis was conducted *a priori*. The aim was to recruit as many participants as possible within the budget limits. Data was collected in June 2015.

#### Participants

A total of 277 participants (48.8% female; 57.7% aged 25–44 years old) voluntarily participated in the online study via Mechanical Turk in exchange for $1.50.

#### Procedure and materials

Similar to Experiments 2 and 3, participants were randomized to either a loneliness or non-loneliness condition, and completed the personality filler questions, the writing task, and two manipulation check items (*α* = 0.89). They were then presented an image of the IKEA beige desk with either bright or dark background lighting as in Experiment 1 (Figure 1). In order to engage participants in a conscious information processing of the object image, participants were asked to write down any thoughts that came to mind when looking at the object image. They were told that an acceptable response should be a minimum of two sentences long, but could be longer. Participants then answered questions about their emotions associated with the object image on the PANAS (Watson et al., 1988), and indicated their overall negative and positive emotions toward the object with two single item ratings (1 = *not at all*; 7 = *very much*). Preference was also rated for the desk using the same four-item scale (α = 0.97) from Experiments 1–3.

### Results and discussion

After receiving the state loneliness manipulation, participants in the loneliness condition reported significantly higher level of loneliness (*M =* 3.22, *SD* = 1.77) than their counterparts (*M* = 2.58, *SD* = 1.75; *t* (275) = 9.17, *p* < 0.01).

The results of the ANOVA testing the effects of loneliness on product preference as a function of product background lighting after participants consciously thought about the object showed no significant main effects of loneliness (*F* (1, 273) = 0.06, *p* = 0.80), or background lighting (*F* (1, 273) = 0.092, *p* = 0.76), and no significant interaction effect (*F* (1,273) = 0.025, *p* = 0.87). This provided initial support for our hypothesis that object presented in dark background lighting evoke an automatic and non-conscious conceptual metaphor of loneliness that drives object preferences for lonely people. We then conducted several ANOVA tests with each PANAS item, and the overall negative emotion and positive emotion as the dependent variables to examine the effects of loneliness, background object lighting, and their interaction on emotions associated with the object. Consistent with our hypotheses, there were no significant main effects or interaction effects, for any of the emotion ratings (*p’s* > 0.30). Together these findings indicate that engaging in conscious information processing of the object’s background lighting eliminates the effects of loneliness on object preference and negative object emotions. This supports our proposition that background darkness evokes a conceptual metaphor of loneliness via automatic, unconscious processing, which in turn influences object preferences.

## Study 5

In the previous Studies we found that loneliness was associated with a preference for objects with bright versus dark background lighting, which is consistent with our hypothesis that objects with a dark background lighting activate a negative self congruity, and therefore motivate a preference for objects with a bright background lighting as a way to avoid a negative self schema and enhance self-esteem [i.e., to create an ideal-self congruity, Sirgy (1985)]. In Study 5 we aimed to more directly test the relevance of the object for one’s self-image by examining the potential moderating role of product authorship, that is, the extent to which the effect of loneliness on object preference depended upon whether the object was being purchased for oneself or for others. If, as we propose, loneliness motivates preferences for objects in bright versus dark background lighting because the dark lighting highlights something negative about the self and creates a negative self-congruity, then this preference should only be present for products that are being considered for oneself, rather than for someone else. To test this, we used a t-shirt, a coffee mug, and a toothbrush as the objects under consideration. T-shirt is an object that is worn, whereas a coffee mug and a toothbrush are objects that are not. These items were also chosen as they can be considered “personal” objects that one might use on a daily basis, and because they were also easily handleable, unlike the large furniture pieces used in the previous studies. We chose two different classes of objects to test whether the expected effects of object authorship on lighting preference differed as a function of wearability, with wearability serving as a proxy for self-referent saliency.

### Method

Approval from the Bishop’s University Research Ethics Committee (Canada) was received prior to data collection. Participants’ consent was obtained online via indicating agreement to an online consent form administered prior to data collection. No power analysis was conducted *a priori*. The aim was to recruit as many participants as possible within the budget limits. Data was collected in June 2013.

#### Participants

A total of 298 students (47.6% female; Mean age = 20.3, *SD* = 3.10) at a Canadian university voluntarily participated in the study in exchange for $3.

#### Procedure and materials

**Figure 4:**
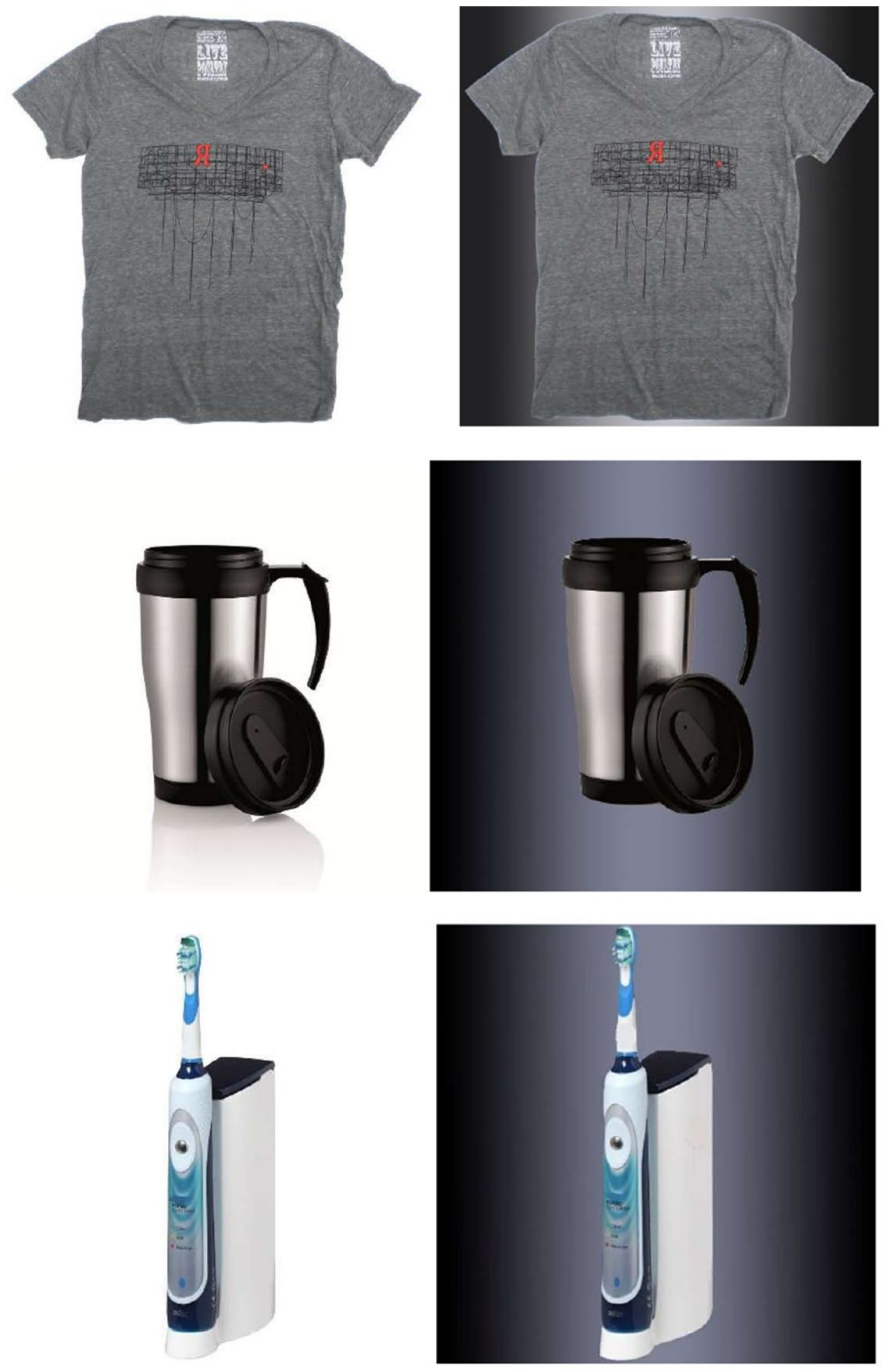
T-shirt, coffee mug, and electronic toothbrush presented in Study 5 with bright and dark background lighting.

Participants first completed the personality filler questions, and the 20-item revised UCLA loneliness scale (*α* = 0.92) (Russell et al., 1980). They were then presented an image of a gender-neutral T-shirt, a coffee mug, and an electric toothbrush (Figure 4) with either dark or bright background lighting, and told to imagine that they were shopping the products either for themselves or for someone else. Participants indicated their preference toward the T-shirt across a five-adjective checklist, with response range from 1 (*bad, unpleasant, negative, unfavorable,* and *dislike*), to 7 (*good, pleasant, positive, favorable*, and *like*; α = 0.96).

### Results and discussion

To test our hypotheses, we conducted a regression analysis with preference toward the three respective products as the dependent variable, and loneliness (mean-centered), authorship (self vs. others), background lighting (bright versus dark), and all their interactions as predictors.

Results for the t-shirt revealed a marginally significant main effect of authorship (*β* = 0.457, *t* = 1.95, *p* = 0.07), a significant two-way interaction between authorship and loneliness (*β* = 0.045, *t* = 2.46, *p* < 0.05), and a significant three-way interaction between authorship, loneliness, and product background lighting (*β* = −0.066, *t* = 2.07*, p* < 0.05). We then conducted spotlight analyses at 1 standard deviation above and below the mean of loneliness scores to examine the interaction between authorship and object background lighting. A total of 204 participants (68.5% of the sample) fell within the ±1 SD range (SD = 11.35). When shopping for oneself, lonely participants reported higher preference toward the t-shirt when the background lighting was bright than when it was dark (*β =* 0.408, *t* = 2.13, *p* < 0.05), whereas non-lonely participants’ preference toward the t-shirt was not impacted by its background lighting (*β* = 0.10, *t* = 0.65, *p* = 0.52). When shopping for others, both lonely participants (*β* = −0.359, *t* = 1.28, *p* = 0.18) and non-lonely participants (*β* = 0.259, *t* = 1.14, *p* = 0.21) showed equal levels of preference when the product background lighting was bright and dark.

Results for the coffee mug and the toothbrush revealed no significant main effects (*p =* 0.93 and *p* = 0.11, respectively) nor any two-way or three-way interactions (*p* > 0.15).

## Study 6

In Study 5 we found that lonely participants demonstrated a preference toward a T-shirt with bright background lighting vs. dark background lighting, but only when it was considered as a purchase for themselves rather than for others. However, there were no similar preferences for background lighting for objects that were not wearable. In this study we sought to replicate these effects, but with different objects – a water bottle and a pair of sunglasses, with a UK rather than North American sample, and a short versus long measure of loneliness. Similar to the T-shirt in Study 5, sunglasses are an object that are worn, whereas a water bottle is an object that is not. As in Study 5, these two objects allowed us to test whether the expected effects of object authorship on lighting preference differed as a function of wearability, with wearability serving as a proxy for self-referent saliency.

### Method

Approval from the University of Sheffield Research Ethics Committee (UK) was received prior to data collection for this between-subjects study. Participants’ consent was obtained online via indicating agreement to an online consent form administered prior to data collection. We aimed to recruit 300 participants as this was the most we could recruit within the budget limits for the study. Data was collected in April 2022.

#### Participants

A total of 296 adults (47.6% female; Mean age = 20.3, *SD* = 3.10) recruited via Prolific participated in exchange for £1. Prolific is a UK-based crowd-sourcing recruitment platform. Five participants were dropped from the data analysis due to the unreasonably short time spent on the survey.

#### Procedure and materials

**Figure 5:**
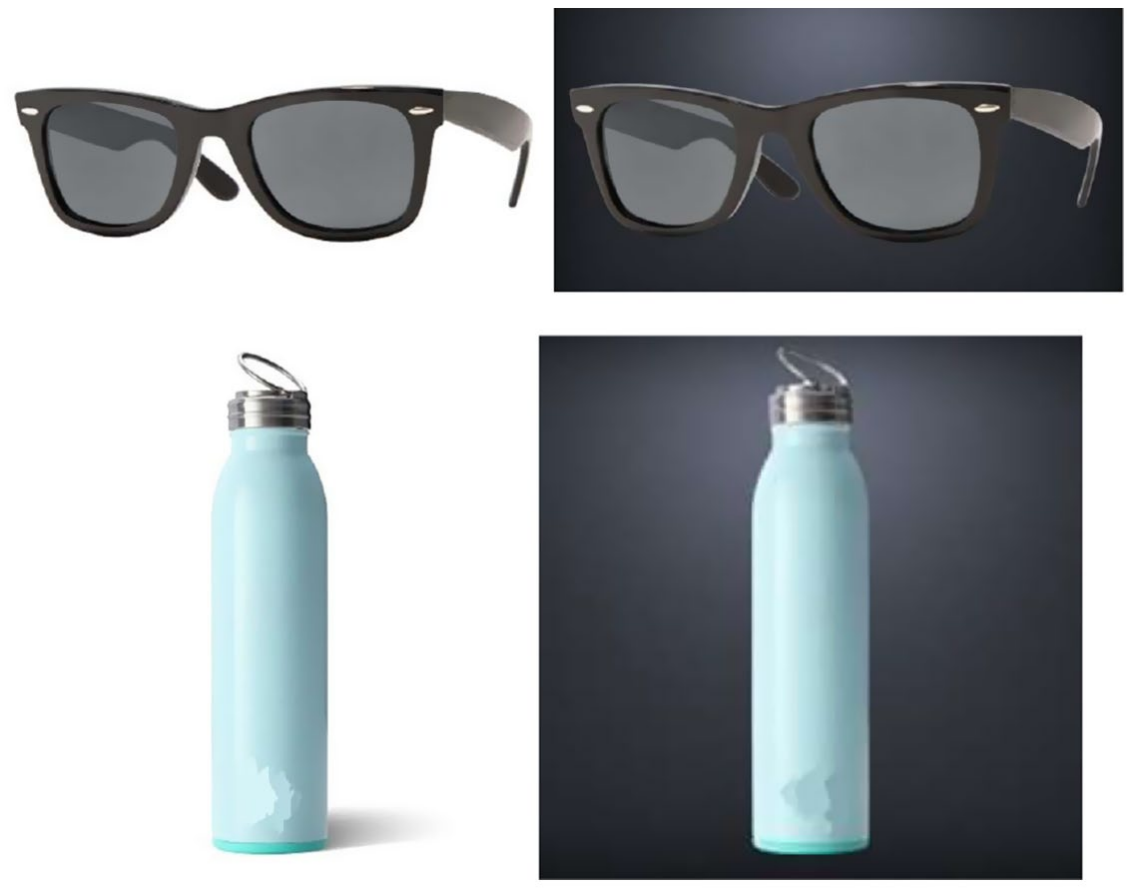
Sunglasses and water bottle presented in Study 6 with bright and dark background lighting.

Participants first answered the personality filler questions, and the 8-item revised UCLA loneliness scale (*α* = 0.78) (Russell, 1996). They were then presented the images of a pair of sunglasses and a water bottle (Figure 5) with either dark or bright background lighting and told to imagine that they were shopping for the products either for themselves or for someone else. Participants indicated their preference toward the products across a five-adjective checklist, with response range from 1 (bad, unpleasant, negative, unfavorable, and dislike), to 7 (good, pleasant, positive, favorable, and like; α = 0.96 and 0.92). They also rated their emotional perceptions (socially isolated, cold, extroverted, warm, sophisticated) of the featured product on a 7-point scale (1 = not at all; 7 = very much; α = 0.53). Then, they indicated their purchase intention measured by two items (“how likely are you to buy this product for yourself (vs. other)” and “how appealing is this water bottle to you (vs. other),” α = 0.96 and 0.93).

### Results and discussion

This study was pre-registered on the Open Science Framework (OSF).^1^ To test our hypotheses, we first replicated the analyses from Study 5 and conducted a regression analysis with preference toward the two consumer products (sunglasses and water bottle) as the dependent variable, and loneliness (mean-centered), authorship (self vs. others), background lighting (bright versus dark), and all their interactions as predictors.

Results for the sunglasses revealed a significant main effect of authorship (*β* = −0.97, *t* = −5.85, *p* < 0.01), a marginal significant three-way interaction between authorship, loneliness, and product background lighting (*β* = −0.94, *t = −1.93*, *p = 0*.05). Results for the water bottle did not reveal any significant main effect of loneliness (*β* = −0.15, *t = −*1.35, *p* = 0.18) or authorship (*β* = −0.08, *t = −*1.32, *p =* 0.19), nor any significant two-way or three-way interaction effects (*p* > 0.40).

We then conducted spotlight analyses for the sunglasses at 1 standard deviation above and below the mean of loneliness scores to examine the interaction between authorship and object background lighting. A total of 185 participants (63.6% of the sample) fell within the ±1 SD range (SD = 0.69).”When shopping the sunglasses for oneself, lonely participants reported marginally higher product preference when the background lighting was bright than when it was dark (*β* = 0.21, *t* = 1.75, *p* < 0.10), whereas non-lonely participants reported no significant difference in terms of product preference toward the sunglasses when the background lighting was dark than when it was bright (β = −0.19, *t* = −1.52, *p* = 0.13). When shopping the sunglasses for others, neither the lonely participants (*β* = −0.05, *t* = −0.46, *p* = 0.65) nor the non-lonely participants (β = 0.07, *t* = 0.59, *p* = 0.56) demonstrated a significant difference in terms of product preference when the product background lighting was bright vs. dark.

We repeated the analysis by conducting a regression analysis on a new dependent variable - purchase intention toward the two consumer products (sunglasses and water bottle), with loneliness (mean-centered), authorship (self vs. others), background lighting (bright versus dark), and all their interactions as predictors.

Results for the sunglasses revealed a significant main effect of loneliness (*β* = −0.20, *t* = −2.23, *p* < 0.05), a significant main effect of authorship (*β* = −0.52, *t* = −10.57, *p* < 0.01), a significant two-way interaction between authorship and loneliness (*β* = 0.19, *t* = 2.0, *p* < 0.05), and a significant three-way interaction between authorship, loneliness, and product background lighting (*β* = −0.31, *t =* −3.33, *p* < 0.01). Results for the water bottle only revealed a significant main effect of authorship (*β* = −0.17, *t* = −2.97, *p* < 0.01), and no other significant main effect or interaction effects were found (*p*’s > 0.05).

We then conducted spotlight analyses at 1 standard deviation above and below the mean of loneliness scores to examine the interaction between authorship and object background lighting. When shopping for oneself, lonely participants reported higher purchase intentions toward the sunglasses when the background lighting was bright than when it was dark (*β =* 0.28, *t* = 2.35, *p* < 0.05), whereas non-lonely participants reported higher purchase intention toward the sunglasses when the background lighting was dark than when it was bright (*β* = −0.25, *t* = −2.1, *p* < 0.05). When shopping the sunglasses for others, both lonely participants (*β* = −0.15, *t* = −1.38, *p* = 0.17) and non-lonely participants (*β* = 0.17, *t* = 1.52, *p* = 0.13) did not demonstrate a significant difference in terms of purchase intentions when the product background lighting was bright vs. dark.

## General discussion

The findings from the six experiments advance research on the understanding of individual differences in loneliness and its metaphorical mapping by extending prior findings regarding the influence of loneliness on personal preferences for temperature to the visual domain. We demonstrate for the first time that loneliness, whether chronic or momentary, is associated with a preference for objects presented with bright rather than dark background lighting, and that this preference is linked to avoidance of negative self-schemas. Consistent with embodied cognition (Barsalou, 2008), conceptual metaphor theory (Lakoff and Johnson, 1980), self-product congruency theory (Sirgy, 1982, 1985), evolutionary views on loneliness (Cacioppo and Hawkley, 2009; Cacioppo et al., 2014), and evidence linking loneliness to avoidance motivations (Cacioppo et al., 2006; Mehrabian, 1994), we found that negative feelings toward objects in dark background lighting, rather than positive feelings toward objects in bright background lighting, explained why people who felt lonely preferred objects presented in bright rather than dark background lighting. We also provide preliminary evidence that the activation of the metaphoric link between loneliness and object preference may involve an information processing route that is unconscious rather than conscious. That the effects of loneliness on object preference occurred only when the object had self-referent salience, further suggests that lonely people’s preferences for objects with bright but not dark background lighting may be motivated by avoidance of a negative self-schema activated by a congruity between the actual lonely self and the object (Sirgy, 1985). This is consistent with the notion that lonely people are sensitive to threatening stimuli in the environment (Cacioppo et al., 2014; Cacioppo and Hawkley, 2009), and that by avoiding objects associated with a threatened aspect of identity (darkness = loneliness), lonely people are able to shield themselves from negative self schemas and thus view the self more positively (White et al., 2012).

The current findings add to our understanding of loneliness and its consequences in several important ways. Research has established that lonely people pay greater attention and are more sensitive to the social aspects of their environment (Cacioppo et al., 2009), especially when these signal threat (Cacioppo and Hawkley, 2009). Our findings further suggest that lonely people may also be sensitive to non-social aspects of the environment and those within the environment that may trigger threats to self-image, such as objects shown in dark background lighting Given this, it is possible that lonely people could also be sensitive to other subtle, non-social aspects of the environment that may metaphorically reflect a threat to self-image by activating self-schemas of being someone who is lonely. Rather than being drawn to objects in dark background lighting, which according to self-product congruency theory (Sirgy, 1982, 1985) would maintain self-consistency, people who feel lonely instead are drawn to objects with light background lighting which reflect an ideal self-congruity and viewing oneself in a more positive light. This motivation for self-enhancement is also self-protective as it helps them avoid the negative and perhaps threatening self-schema of viewing oneself as someone who is lonely,

It is also possible that situational darkness may also influence how lonely people see themselves in other ways. For example, there is some evidence that even in the absence of social cues in the environment, situational darkness can induce self-construals that are more interdependent (Steidle et al., 2013). For the individual who is lonely, the promotion of interdependent self-construals that occur from the perception of environmental darkness could therefore further heighten awareness of feeling lonely. Specifically, darkness as a prompt for construing oneself in relation to others could highlight the lonely individual’s lack of desired social connections, which could further induce avoidance of objects presented in environmental darkness. Research specifically examining this self-construal explanation would be an interesting and potentially fruitful area that could yield additional insights into the embodied effects of loneliness within the visual domain.

The current findings also contribute to current knowledge regarding the effects of loneliness on people’s judgments and decisions (e.g., Zhong and Leonardelli, 2008) by demonstrating that the metaphorical linkage of dark background lighting with loneliness has an influence on people’s preferences. Consistent with self-product congruency theory (Sirgy, 1982, 1985), and sensory marketing (Krishna, 2012; Krishna and Schwarz, 2014), these preferences in turn may impact people’s consumption behaviors in several important ways. Although the effects in the current study were demonstrated with furniture and personal products, it is possible that similar effects could be found for pictures of environmental spaces and not just the objects within them. For example, pictures of rooms, homes, offices, cafes, or other environments shown with dark background lighting, may be viewed as being less desirable by lonely people and impact their decisions to visit, buy, or rent such places. In this respect, our findings offer a caveat for the prevailing view that dark background colors are associated with desirable qualities such as authority, prestige and exclusivity (Gorn et al., 1997) that denote a cool sophistication and a powerful sense of luxury. For lonely people, the dark background lighting associated with these images may instead have the reverse effect of making such objects and spaces seem less, rather than more, desirable, because they highlight a self-congruency between a less desired, actual self who is lonely. Rather than promoting consumer preferences, this dark background may elicit product avoidance and negative attitudes toward the product, or even discourage brand loyalty. In these respects, the current findings highlight several potential fruitful areas of research to better understand the effects of loneliness on people’s judgments, decision making, and behavior.

Although we propose that lonely people have a negative reaction to the objects with dark background lighting because of a bidirectional, dynamic link between loneliness and darkness that amplifies feelings of loneliness (e.g., Bower, 1981; Ingram, 1984) and thus threatens self-esteem, other explanations are possible. From an evolutionary perspective of loneliness (Cacioppo et al., 2006), when a person feels lonely they also feel unsafe, and this activates an *anachronistic survival mechanism* that heightens awareness and sensitivity to potential threats and attacks. The idea of darkness conveyed by dark background lighting implies not being able to see one’s surroundings very well, and may therefore heuristically suggest vulnerability to harm (see Schaller et al., 2003). For a lonely person, background darkness could activate unpleasant feelings of threat more generally, rather than those specifically related to the self, that reduce preferences for images of objects, spaces, and products with dark background lighting. However, Studies 5 and 6 provided evidence that the preferences for objects in bright background lighting were only present when the object was framed as being for oneself rather than for others, and when the object was wearable, and therefore had high self-referent saliency. This suggests lonely people may be more sensitive to objects that highlight specific threats to the self rather than general threats in the environment. Research testing these alternative explanations would provide further support for the explanations we propose.

Lastly, it is possible that the effects of background lighting on product preferences are due to the greater or lesser contrast created by presenting objects against light or dark backgrounds. From this perspective, the preferences may be grounded in a liking of greater contrast and/or of a lack of visible backgrounds that could be represented by having a light versus dark object background. However, research on the effects of environmental darkness on psychological states in the context of dark tourism provides evidence that counters this alternative explanation. Across four different studies, Lv et al. (2022) found consistent effects of simulated (two-dimensional) and actual (three-dimensional) visual darkness on psychological states. Specifically, the effects on psychological states (i.e., darkness experiences) due to darkness versus lightness of the photo, or whether the photo was presented against a light or dark background, were consistent with each other and with those from actual ambient darkness. Although this evidence does not explicitly rule out the above alternative explanation, it does provide compelling evidence that visual embodied effect of darkness may be unified across different dimensions, forms, and expressions (Lv et al., 2022), such as those employed in the current research.

### Limitations and strengths

The current research should be considered in the context of several limitations and strengths. First, although we presented different objects in background lighting, it is unknown if the effects found will generalize to other object types and classes; they could be particular to the objects tested. Future research is needed to address this issue. Although the effects of product authorship on lighting preferences were tested for five different objects, two wearable and three not, further research on different objects with varying degrees of self-reference is needed to understand the extent to which the effects found in the current set of studies generalizes to other objects. One could also argue that the furniture used in Studies 1 and 2 are not technically “wearable” and so finding similar effects as for the wearable objects may calls into question this explanation. However, in Studies 5 and 6 objects were chosen because they were personal in nature, unlike the generic and impersonal nature of the furniture used in the initial studies. From this perspective, the categorization of wearable versus not is embedded within the broader category of personal or not. That is, for personal objects, those that are not wearable may hold less self-referent salience than those that are wearable, whereas for impersonal objects, object preferences may be independent of wearability and depend more on background lighting. Further research exploring this proposition would be useful to clarify the influence of impersonal and personal objects on the preferences of people who are lonely.

Lastly, dark background lighting was represented as a backdrop within a picture rather than as environmental ambient lighting in the current study. Although we did not directly test whether the presentation of objects within a room with the lights dimmed would have the same effects on the preferences of lonely people, previous research using this approach to examine the psychological effects of differences in sensory lightness and darkness found that it was a reasonable proxy for the psychological effects of changes in levels of three-dimensional visual lighting changes (Lv et al., 2022). It is therefore possible that the effects of three-dimensional lighting on the preference of lonely people are stronger than those found using two-dimensional visual lighting changes, and that the current study underestimated these effects. Previous research has also demonstrated that hopelessness, a state associated with loneliness (Joiner and Rudd, 1996), is linked to changes in perceptions of the brightness of environmental lighting, which could influence lighting preferences (Dong et al., 2015), and that darkened ambient room lighting led people to feel more disconnected from others (Huang et al., 2018). Taken together, this research supports the idea that loneliness would also influence preferences in situations of actual rather than simulated ambient lighting.

Despite these limitations, the current set of studies has clear strengths including replicating the results with both chronic and state loneliness, and with large community and student samples. Ruling out the competing hypotheses of object color in Studies 1 and 2 provides additional support for the hypotheses that the effects were due to the background lighting. In addition, testing and ruling out the alternative hypothesis that the effects found were due to depression rather than loneliness increases confidence in the validity of the findings. Replicating the findings from Study 5 with objects that were and were not wearable in Study 6 provided further insights into how object authorship impacts the lighting preferences associated with loneliness. Lastly, partially replicating the findings from Study 5 with a UK rather than a North American sample in Study 6, provides some support for the generalizability of the findings.

## Conclusion

Overall, the present set of studies provide convergent evidence for the metaphorical linkages of loneliness with dark background lighting and the effects of this metaphorical thinking on the preferences associated with loneliness. Whether loneliness was chronic or momentary, we found that people who felt lonely preferred objects presented in bright rather than dark background lighting, and this preference appeared to be motivated by wanting to avoid association with the object in dark background lighting, rather than being drawn to the object in the bright background lighting. Together, these findings support the proposition that objects that evoke a conceptual metaphor of loneliness activate undesired self-schemas for lonely people, which in turn motivates less preference for the lonely object in favor of the non-lonely object. With an ever-increasing number of people in society feeling lonely (Office of National Statistics, 2025), it is important to understand the ways in which loneliness might influence people’s judgments, decisions, and behavior. Our findings make a novel contribution to this understanding and suggest avenues for further investigation.

## Data Availability

The raw data supporting the conclusions of this article will be made available by the authors, without undue reservation.

## Footnotes

1
https://osf.io/gxth4/?view_only=a972d3c6f4ba4288a3449436d34f015c
